# Antimicrobial resistance (AMR): an important one health issue for layer and meat poultry industries worldwide

**DOI:** 10.1016/j.psj.2024.103690

**Published:** 2024-04-04

**Authors:** Chris J. Morrow

**Affiliations:** ⁎ School of Veterinary Science, The University of Melbourne, Parkville, Victoria 3008, Australia; † Bioproperties, Ringwood, Victoria 3134, Australia

**Keywords:** AMR, mycoplasma control, antibiotic use in lay, one health

## Abstract

Routine antibiotic administration has been used in intensive animal industries for a long time for health and production benefits. There is now a concerted effort to limit antibiotics administration to only treatment of clinically affected animals and to look for other alternative solutions combined with better husbandry practices for the benefits routine antibiotic administration seems to provide in intensive farming systems. In this paper it is argued that the benefits from routine antibiotics in chickens administration in lay are from suppression of the effects of mycoplasma infections. Mycoplasma freedom has been recommended but is not always practical. Vaccination of mycoplasma negative chickens with live mycoplasma vaccines is now being used (with biosecurity) to decrease antibiotic dependence in lay of poultry in many parts of the world.

## INTRODUCTION

Intensive livestock industries are the largest users of antibiotics (73%) in the world (van Roeckel et. al., 2019). The realization of potential problems of the induction of antibiotic resistance in animal pathogens (largely an animal health problem) and other bacteria (potentially part of the human health problem AMR) that are exposed to the antibiotics is becoming more appreciated and has become a major one health issue (George, 2019). There have been concerted efforts to reduce this use especially focusing on the use of antibiotics at ostensibly subtherapeutic levels as growth promoters. More recently there are also general pressures to decrease other antibiotic use in food producing animal industries considering AMR consequences of antibiotic use when deciding to medicate. In chickens, it is argued here that the consistent benefits from traditional antibiotics programs in the laying production period is from their prevention of the chronic effects of mycoplasma infections in treated birds and their progeny (including production inefficiencies and prevention of diseases).

An analysis of where and why antibiotics are used in poultry production systems allows more focused reductions in routine administration of antibiotics when alternative strategies are identified. The major use of routine antibiotics in layers and broiler breeders in production are programs where antibiotics (initially tetracycline or tylosin) are given every 4 to 8 wk for a week in feed (Kleven 2008). The antibiotics currently used are much more varied including macrolides but invariably have antimycoplasmal activity. In a recent survey of 14 countries in Asia, 48 out of 77 grandparent broiler breeder flocks surveyed used routine antibiotics to prevent disease and 40 out of 85 parent stock breeder flocks used antibiotics (M. Charles, personal communication. 2023, see supplementary materials). The routine uses of antibiotics in lay is wider than just Asia and is more common in commercial layers and layer breeders especially with antibiotics with zero withdrawal times.

The quantities of antibiotics fed to birds in lay is not widely appreciated outside manufacturers, suppliers and users. Although a week-a-month (**WAM**) tetracycline or tylosin preventative programs are reported (Kleven, 2008) it is difficult to find information on how much antibiotic is used in lay but in an estimate that using a WAM regime that one million layers would use 3.65 tons of antibiotic active per year. A broiler breeder operation consisting of 400K parent stock would use 6.7 tons of antibiotic active per year on the assumption that the broilers produced would also receive three days of preventative antibiotics starting at d 18 to prevent “post vaccinal reaction” (Morrow, 2021). A sensitivity analysis looking at longer periods (say 8 wk) between treatments, lower treatment rates or shorter treatments still gives total yearly quantities of active use in tons. It should be noted that over time there is a tendency for “dosage creep” to occur where in field dosage levels to achieve effective responses may increase up to tenfold. In the past antibiotic residues have largely been a problem for broiler products especially when exported but now commercial eggs are also receiving attention (Ma et al., 2022).

Continuous feeding of antibiotics in the laying period have been used this way since the 1960s (Brackett et al., 1960) and indeed many of the clinicians now prescribing antibiotics are not aware that the main targets of this treatment are avian mycoplasmas *Mycoplasma gallisepticum* (**MG**) and *M. synoviae* (**MS**)*.* There is a belief amongst some clinicians that the antibiotics are generally dampening down the impact of “bacteria” on chicken production and not targeting any specific bacteria.

Here it is argued that the target giving the benefits are pathogenic mycoplasma as these infections are chronic active infections of the birds for life. This beneficial effect documented are an increased egg output and a better feed conversion into eggs in MG infected layers continuously treated with tylosin throughout the total laying period (Ose et. al., 1979). Routine administration of antibiotics in lay targeting bacteria cell wall synthesis has not been observed in the experience of the author.

Antibiotic resistance in Mycoplasma is emerging as an issue in South-East Asia (Morrow et al., 2020, Achari et al., 2023) and this is driving increases in dosages and trial of new antibiotics or antibiotic strategies (combination therapy for example). Antibiotic resistance in avian mycoplasma is probably only a problem for avian mycoplasma control rather than a problem of resistance transfer from these mycoplasmas to other pathogens. However, the use of antibiotics also puts pressure to select for antimicrobial resistance (**AMR**) in the chicken's microbiota and this AMR is important for Salmonella and Campylobacter from the zoonotic perspective and perhaps some ESKAPE organisms (Denissen et al., 2022, Salem et al., 2024).

The WAM antibiotic treatment of breeders could be influencing resistance patterns in the broiler generation. *Campylobacte*r spp. appear to be commensals in the chicken and not subject to control programmes (except in some Scandinavian countries) and physicians like having (effective) macrolides to treat affected humans (Trott et. al., 2021) highlighting the need to consider resistance development here. Campylobacter contamination of broiler carcasses is well studied but the effect of the use of antibiotics in lay in broilers and direct contamination of eggs is poorly understood (Cox et. al., 2012). Campylobacters are in young broilers (Colles et. al., 2021) and presumably some of these strains come from the parents. Zoonotic Salmonella can be attacked by different strategies (freedom of specific serotypes) including Salmonella vaccination.

Although microbiologists always point out that antibiotic resistance could be natural or intrinsic, the continual pressure of antibiotics on the microbiota could increase incidence and drive the development of novel resistance configurations. The antibiotic resistance in the poultry microbiota induced by antibiotics in lay could be a potential problem for humans by the contamination of meat and eggs.

The cessation of routine antibiotic programs should see the decrease of resistance in chicken microbiota. An interesting example is the loss of erythromycin resistance in the commensal *Enterococcus fecium* in Australian poultry isolates over the last 20 years paralleling decreased antibiotic usage due to mycoplasma control by vaccination and phasing out multiage breeder farms (O'Dea et. al., 2019). To get these benefits it was important to control both MG and MS by vaccination (or freedom) and biosecurity.

It was appreciated very early on that farming mycoplasma negative stock is the best strategy to prevent mycoplasmal associated diseases. The maintenance of large mycoplasma negative poultry populations with no resistance to challenge often is more risk than the producer is willing to take (Kleven 2008, Bradbury and Morrow 2021).

Alternative strategies for controlling avian mycoplasma infections in lay include sourcing mycoplasma free replacement stock and using live mycoplasma vaccines (Bradbury and Morrow 2021). Mycoplasma free replacement stock is becoming increasingly available in many parts of the world and strategic vaccination using attenuated live vaccines appears to decrease field strain populations. This area needs further investigation.

It is time to phase out the routine use of antibiotics in lay in breeders and layers and consider antibiotic use only in cases of clinical mycoplasmosis confirmed by laboratory diagnosis (**PCR**) and knowledge of local mycoplasma strains resistance patterns, aligning with One Health antimicrobial stewardship. We need to keep access to antibiotics for the treatment of clinically affected flocks on welfare grounds but we need to prevent mycoplasma field strains from impacting our production systems.

## Appendix Supplementary materials

Image, application 1

Image, application 2

Image, application 3

## Footnotes

Supplementary material associated with this article can be found in the online version at doi:10.1016/j.psj.2024.103690.
