# Supplementary material for: Antimicrobial resistance (AMR): an important one health issue for layer and meat poultry industries worldwide
Source: Poult Sci. 2024 Apr 4;103(7):103690. doi: 10.1016/j.psj.2024.103690 (PMC11063636; doi:10.1016/j.psj.2024.103690)
Supplement: Supplementary file 1 [file mmc1.pdf]

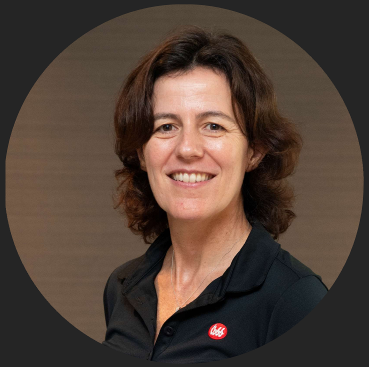

One family. One purpose. Feed the world. Make a difference.

Cobb Health Survey 2023

Dr Magali Charles
Regional Veterinarian Cobb Asia Pacific

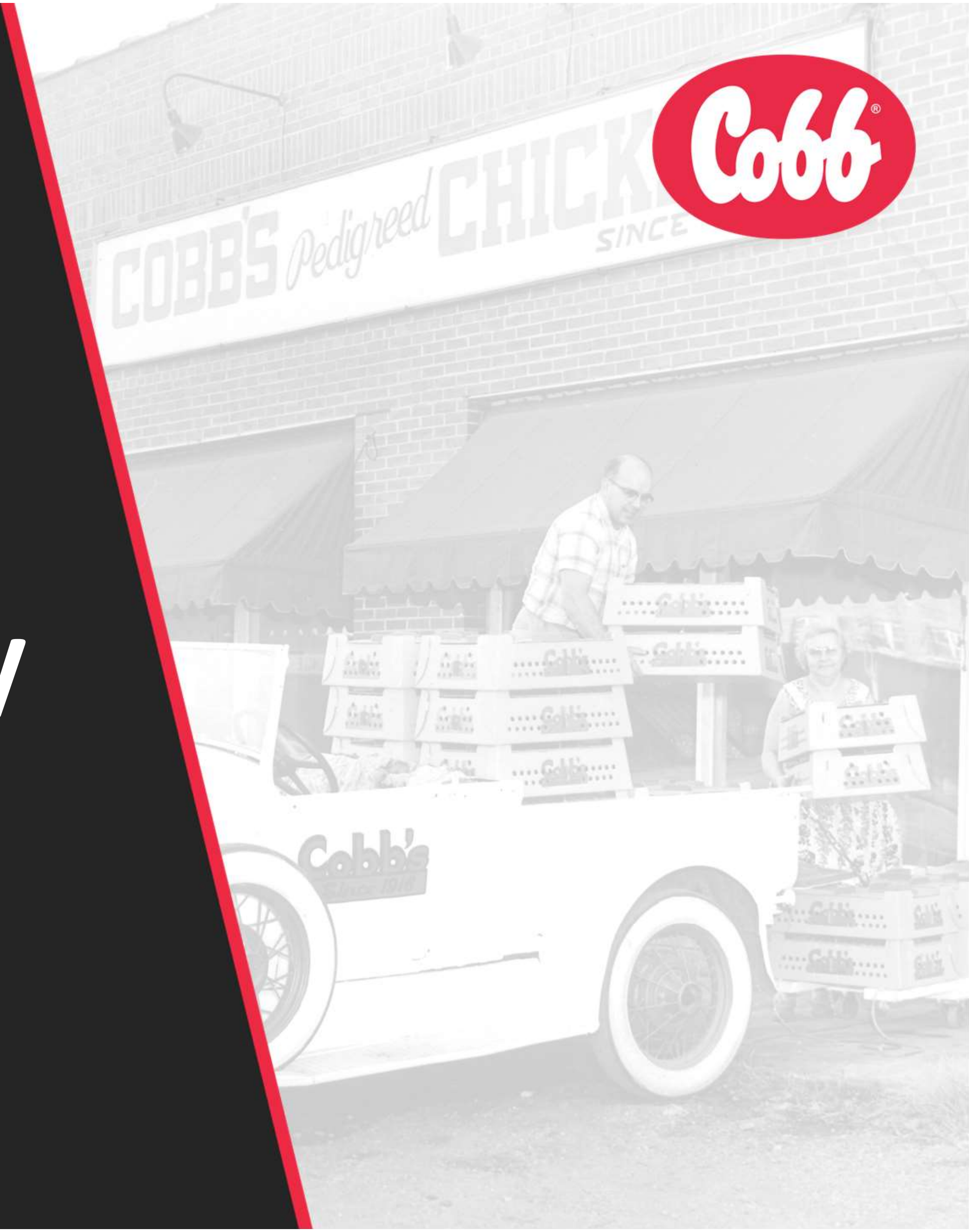

Your concerns are our concerns

- Survey send to 200 Cobb customers and pharmaceutical companies in Asia
- 85 Questions:

What are your principal **concerns** about breeder/broiler health?

- Answers

The answers rely on your concerns and not on the outbreaks that you faced.

High/ medium/ low concern or not a problem at all.

- What are the emerging diseases that you are concerned about?

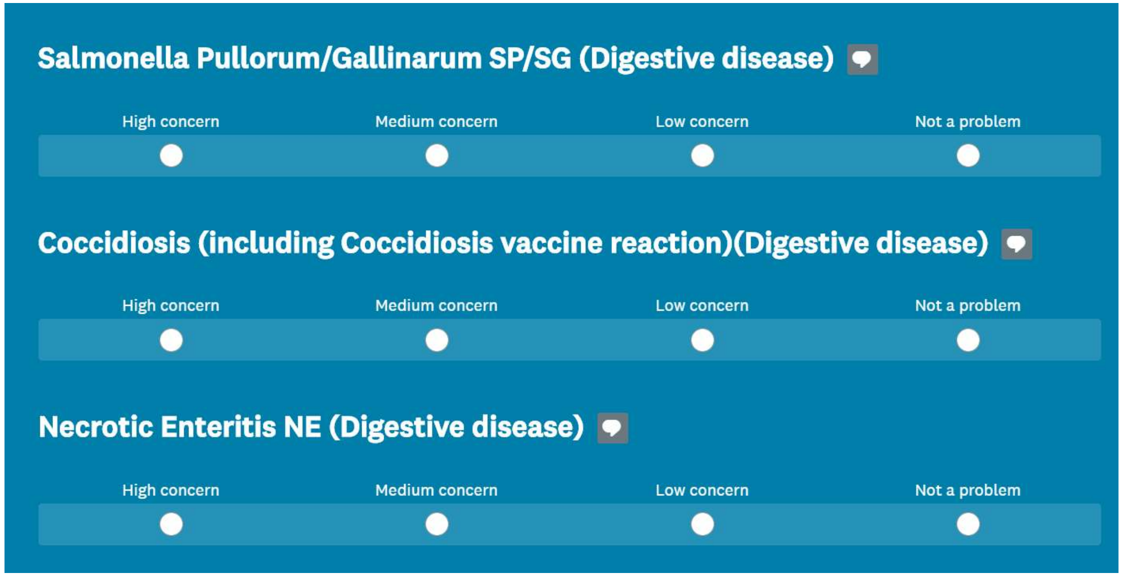

Salmonella Pullorum/Gallinarum SP/SG (Digestive disease)

High concern | Medium concern | Low concern | Not a problem

Coccidiosis (including Coccidiosis vaccine reaction)(Digestive disease)

High concern | Medium concern | Low concern | Not a problem

Necrotic Enteritis NE (Digestive disease)

High concern | Medium concern | Low concern | Not a problem

ONE FAMILY.
ONE PURPOSE.

Participants

- 94 Participants
- 14 Countries
- For all survey participants: individual summary of Asian and own country results
- Answers compiled for Asia: average score answer by country.

  1 country = 1 survey

Country	Participants
Indonesia	18
Philippines	15
China	13
South Korea	10
Malaysia	8
Thailand	7
Bangladesh	7
Nepal	5
Pakistan	2
Vietnam	2
India	2
Sri Lanka	3
Brunei	1
Taiwan	1

ONE FAMILY. ONE PURPOSE.

Threshold

- **Grading**

Weighted scores:

Not a problem = 0 point.

Low = 2 points

Medium = 4 points

High =6 points

- **Interpretation:**

No a problem below 1

Low concern 1 to 3

Medium concern 3 to 5

High concern 5 to 6

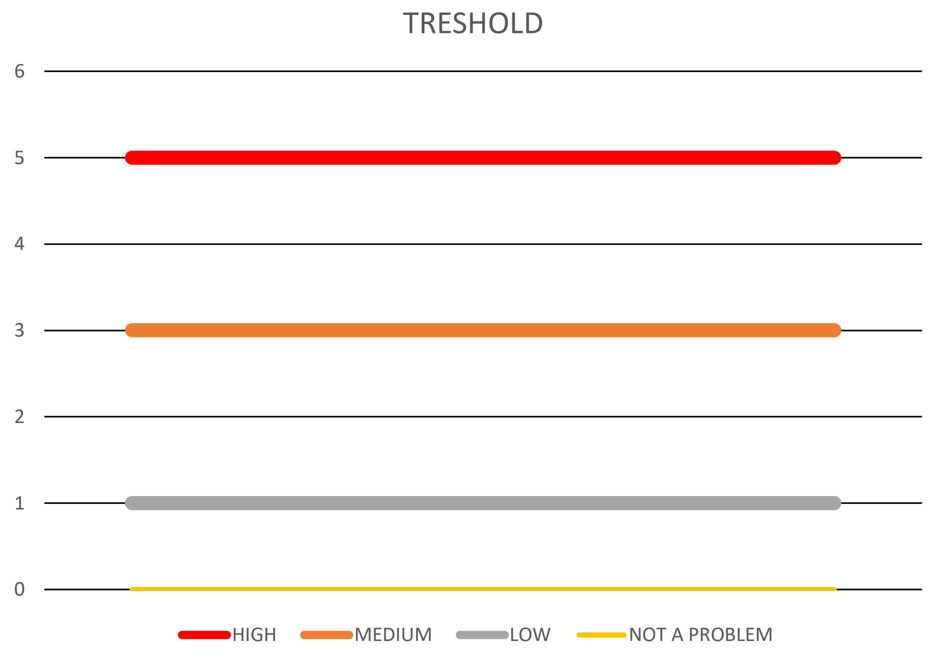

ONE FAMILY.
ONE PURPOSE.

Asia’s main concerns in BREEDERS
TOP 10 DISEASES in 2023

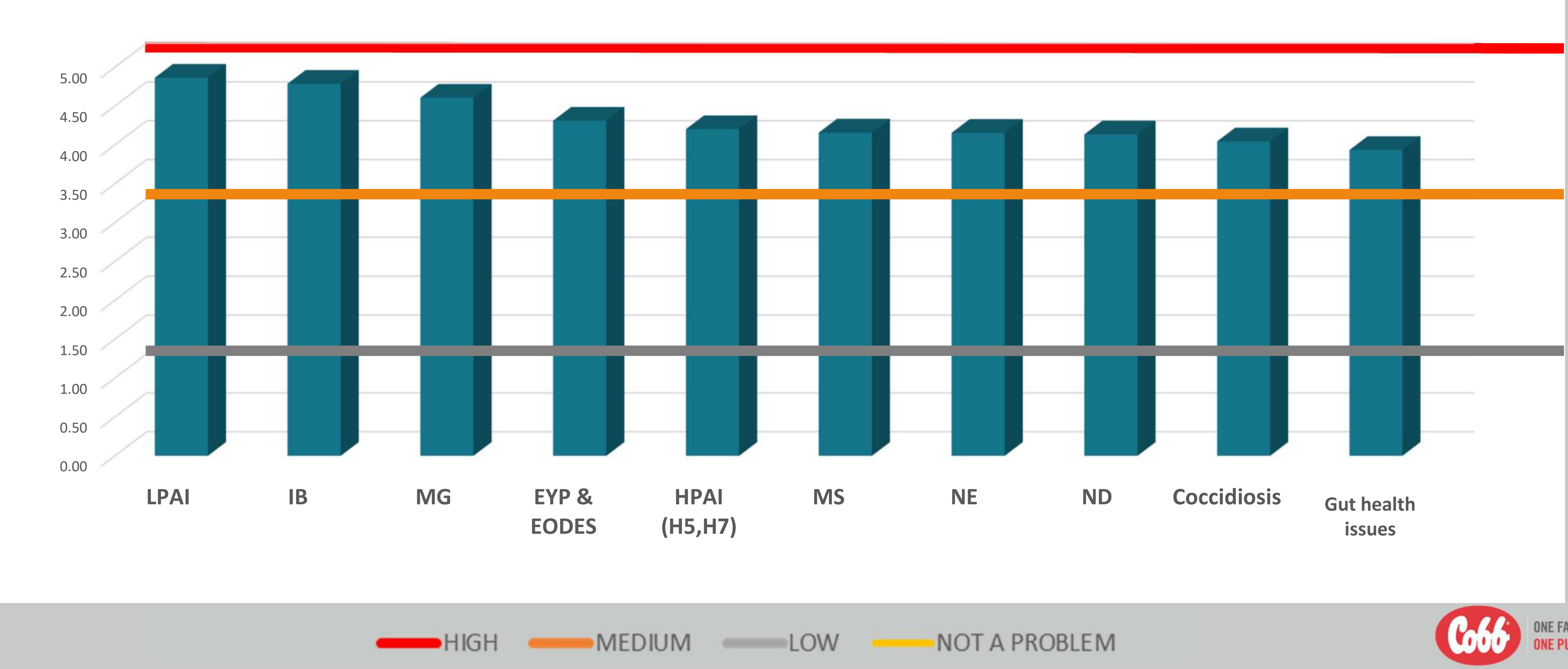

ONE FAMILY. ONE PURPOSE.

Asia's main concerns in BROILERS
TOP 10 DISEASES in 2023

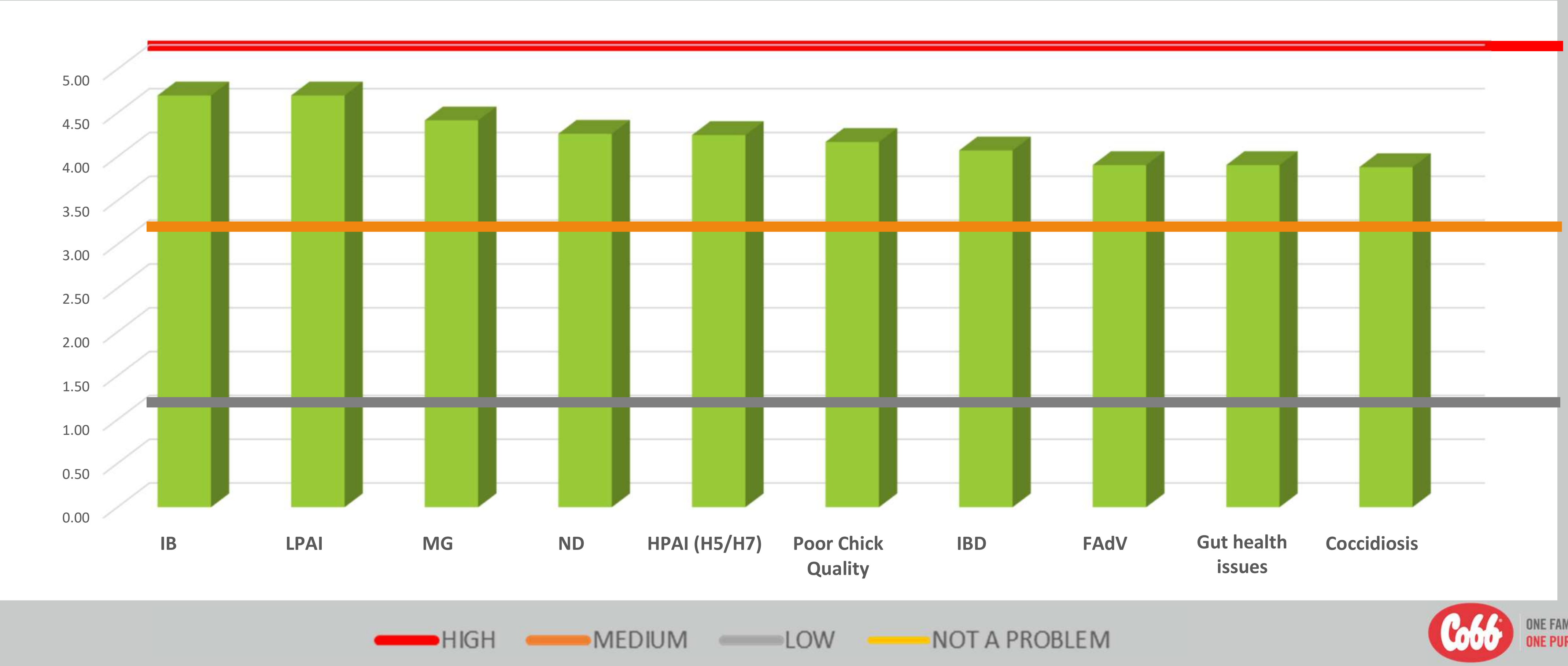

ONE FAMILY.
ONE PURPOSE.

Antibiotic resistance

Alternatives to antibiotics 2023

Have you successfully replaced antibiotics with alternative solutions?

Yes 40% (37/91) No 60% (54/91)

Probiotics, organic acids, vaccines, vitamins, Chinese traditional medicine, essential oil, good quality raw material, bacteriophages, management

Antibiotics 2023

In GP breeder flocks, do you use prophylactic antibiotic treatments to prevent diseases?

Yes 62% (48/77) No 37% (29/77)

Do you use prophylactic antibiotic treatments **in PS breeder flocks** to prevent diseases?

Yes 47% (40/85) No 53% (45/85)

In broiler flocks, do you use prophylactic antibiotic treatments to prevent diseases?

Yes 51% (44/86) No 49% (42/86)

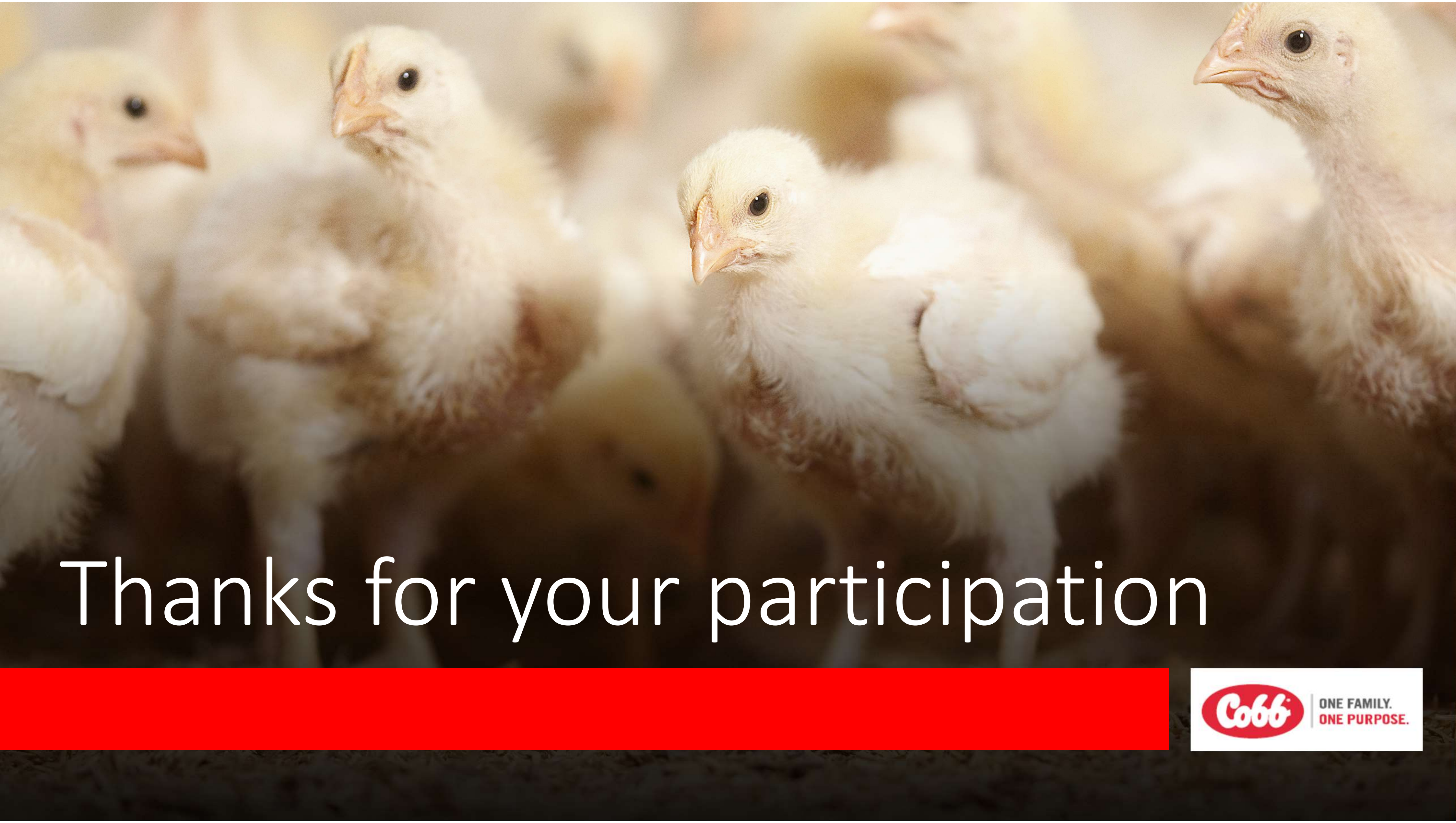
Thanks for your participation
Cobb
ONE FAMILY.
ONE PURPOSE.
