# Supplementary material for: Antimicrobial resistance (AMR): an important one health issue for layer and meat poultry industries worldwide
Source: Poult Sci. 2024 Apr 4;103(7):103690. doi: 10.1016/j.psj.2024.103690 (PMC11063636; doi:10.1016/j.psj.2024.103690)
Supplement: Supplementary file 2 [file mmc2.pdf]

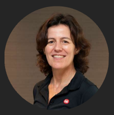

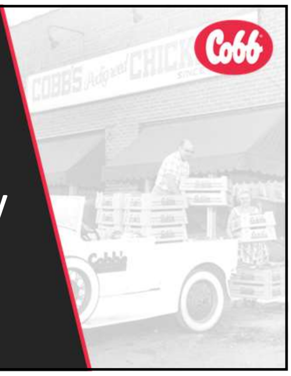

One family. One purpose. Feed the world. Make a difference.

Cobb Health Survey 2023

Dr Magali Charles
Regional Veterinarian Cobb Asia Pacific

1

Your concerns are our concerns

- Survey send to 200 Cobb customers and pharmaceutical companies in Asia
- 85 Questions:

What are your principal **concerns** about breeder/broiler health?

- Answers

The answers rely on your concerns and not on the outbreaks that you faced.

High/ medium/ low concern or not a problem at all.

- What are the emerging diseases that you are concerned about?

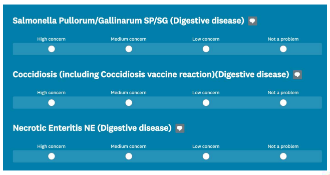

2

Participants

- 94 Participants
- 14 Countries
- For all survey participants: individual summary of Asian and own country results
- Answers compiled for Asia: average score answer by country.

1 country = 1 survey

Country	Participants
Indonesia	18
Philippines	15
China	13
South Korea	10
Malaysia	8
Thailand	7
Bangladesh	7
Nepal	5
Pakistan	2
Vietnam	2
India	2
Sri Lanka	3
Brunei	1
Taiwan	1

3

Threshold

- **Grading**

Weighted scores:
Not a problem = 0 point.
Low = 2 points
Medium = 4 points
High =6 points

- **Interpretation:**

No a problem below 1
Low concern 1 to 3
Medium concern 3 to 5
High concern 5 to 6

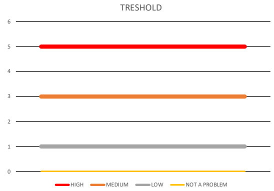

4

Asia's main concerns in BREEDERS TOP 10 DISEASES in 2023

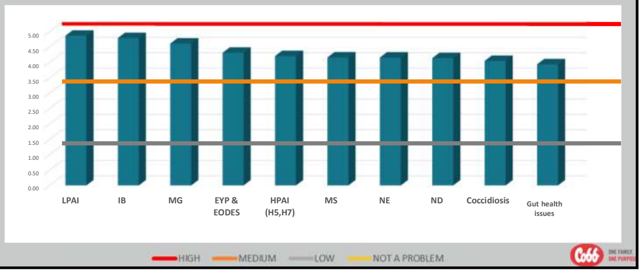

5

Asia's main concerns in BROILERS TOP 10 DISEASES in 2023

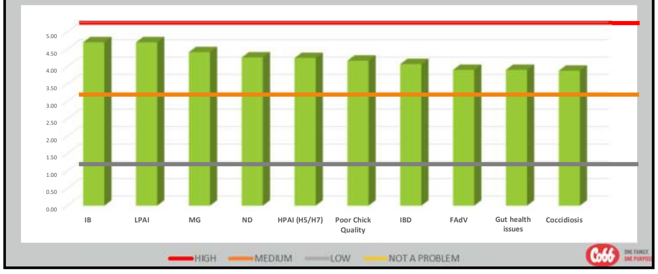

6

Antibiotic resistance

7

Alternatives to antibiotics 2023

Have you successfully replaced antibiotics with alternative solutions?

Yes 40% (37/91) No 60% (54/91)

Probiotics, organic acids, vaccines, vitamins, Chinese traditional medicine, essential oil, good quality raw material, bacteriophages, management

8

Antibiotics 2023

In GP breeder flocks, do you use prophylactic antibiotic treatments to prevent diseases?
Yes 62% (48/77) No 37% (29/77)

Do you use prophylactic antibiotic treatments **in PS breeder flocks** to prevent diseases?
Yes 47% (40/85) No 53% (45/85)

In broiler flocks, do you use prophylactic antibiotic treatments to prevent diseases?
Yes 51% (44/86) No 49% (42/86)

9

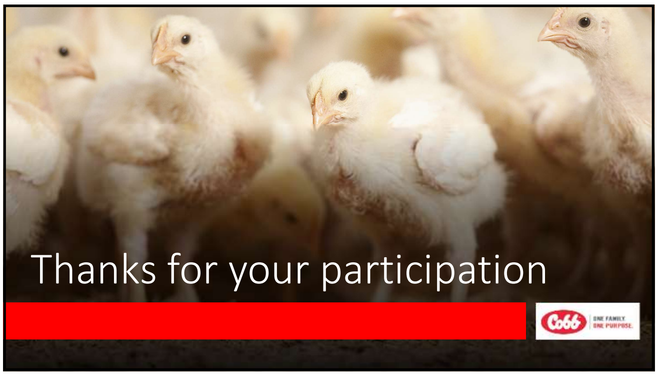

10
